# Liver Injury Induced by High-Dose Methylprednisolone Therapy: A Case Report and Brief Review of the Literature

**DOI:** 10.5812/kowsar.1735143X.713

**Published:** 2011-08-01

**Authors:** Krzysztof Gutkowski, Alina Chwist, Marek Hartleb

**Affiliations:** 1 Department of Gastroenterology and Hepatology, Medical University of Silesia, Katowice, Poland

**Keywords:** Methylprednisolone, Hepatotoxicity, Adverse drug reactions, Multiple sclerosis

## Abstract

Corticosteroids are used widely to treat many types of disease. In general, these drugs are considered safe for the liver; however, recent reports have demonstrated that high-dose methylprednisolone (MT) may cause severe liver injury. Here, we report a case of a 24-year-old female who was given pulsed MT therapy for multiple sclerosis. MT induced icteric hepatitis and impaired liver synthetic function. Hepatotoxicity developed several weeks after drug exposure, and the causal association with MT was confirmed by unintentional rechallenge test. A brief review of the literature on corticosteroid-induced hepatotoxicity is presented.

## 1. Introduction

Adverse drug reactions are frequent and remain underestimated causes of acute liver injury that sometimes lead to liver failure, requiring liver transplantation.

High-dose intravenous glucocorticosteroid treatment is one of the most efficient therapeutic options for severe exacerbations of many autoimmune diseases. A review of the literature shows that corticosteroids are not entirely safe for the liver and have been occasionally linked to severe hepatotoxicity [1][2][3]. We present a case of a 24-year-old woman who was treated with pulsed methylprednisolone (MT) for multiple sclerosis. MT induced serious liver injury, developing as icteric acute hepatitis with impaired prothrombin synthesis. Hepatotoxicity with prolonged latency appeared after the second MT pulse, and the causal relationship between MT and liver injury was confirmed by unintentional rechallenge test.

## 2. Case Report

A 24-year-old woman with a 3-year history of multiple sclerosis and a primary diagnosis of retrobulbar optic neuritis was referred to our department due to severe acute hepatitis. Three months earlier, she was admitted to the neurology department due to exacerbation of multiple sclerosis. Neurological treatment began with a 6-day course (0.5 g/d) of high-dose intravenous MT, resulting in full recovery of her left arm function. Routine laboratory examinations showed normal liver tests and no autoantibodies in the peripheral blood.

The patient was subsequently switched to beta-1b interferon (Betaferon) treatment and received 2 doses of this drug without any immediate or delayed adverse reactions. After 6 weeks, she developed a subsequent flare of MS, presenting as left-sided limb paralysis. A high-dose intravenous MT pulse (total dose 3.0 g) and 2 injections of Betaferon were given, effecting nearly a full recovery. Four weeks after the MT pulse and 1 week after her last Betaferon dose, the patient developed jaundice with elevated serum levels of aspartate aminotransferase (AST) 900 IU/L (n: 10-31 IU/L), serum alanine aminotransferase (ALT) l740 IU/L (n: 9-34 IU/L), serum alkaline phosphatase 186 IU/L (n: 38-126 IU/L), and serum GGTP 50 IU/L (n: -38 IU/L). The serum bilirubin level was 17.9 mg/dL (n: 0.3-1.2 mg/dL), the direct bilirubin was 16.1 mg/dL, and the prothrombin index was 35.1% (INR 2.52). The serum AFP level was 17.18 ng/mL (n: < 5 ng/mL). The patient had no history of hepatic disease and denied any use of alcohol. In the previous 6 months, the patient took acetaminophen (used only in a single dose before each Betaferon injection) and oral contraception, which was stopped immediately before the second MT pulse. Serological tests for hepatitis B (including antibodies to HBc), hepatitis C, hepatitis A, and infection with cytomegalovirus (CMV) were negative. Smooth muscle antibodies (SMA) were found in high titers (> 1:320), and autoantibodies against mitochondria and nuclei were undetected by direct immunofluorescence tests. Her copper urinary excretion was not elevated. An ultrasound abdominal examination showed an intact liver and a normal-sized spleen. No liver biopsy was done due to a low prothrombin level. With a MELD (model for end-stage liver disease) score of 26, the patient was a potential liver transplantation candidate. Fortunately, her liver function improved spontaneously, and the patient was discharged after 3 weeks with normal aminotransferase levels. Considering the potential hepatotoxicity of interferons, we cautiously suspected the Betaferon of inducing liver injury. Three months later, the patient developed a consecutive flare of multiple sclerosis, for which she received a 6-day intravenous MT pulse (total dose 3.0 g) without Betaferon. Her neurological symptoms resolved, but 4 weeks later, she was readmitted to our department with symptoms of acute hepatitis. The serum level of AST was 1488 IU/L, ALT was 1129 IU/L, alkaline phosphatase was 164 IU/L, GGTP was 168 IU/L, prothrombin index was 47% (INR 1.71), and serum bilirubin was 7.3 mg/dL. No drugs, as before, were used to treat the liver disease, and after several weeks, aminotransferases and bilirubin reached reference levels.

## 3. Discussion

We report two serious liver injuries that were related to pulsed MT treatment. The diagnosis was confirmed by the dechallenge-rechallenge relationship between drug administration and hepatitis. Based on a literature review, we identified 12 case reports of corticosteroid-induced hepatotoxicity (Table).

**Table:** Glucocorticosteroid-Induced Hepatotoxicity: Review of 13 Cases

**Reference**	**Age/Sex**	**Principal Disease**	**Type of Steroid**	**Dose and Duration ofTreatment**	**Max. ALT a/AST a , IU/L**	**Max. GGT a /ALP a , IU/L**	**Histology**	**ConcomitantTreatment**	**Follow-up**
Gerolami *et al.* [8]	27/F a	Crohn disease	MP a	50 mg daily IV, 2 days	7.5xN a /3.2xN	5.1xN/1.8xN	Biopsy not done	None	Normalization of liver tests after MP discontinuation
			P a	60 mg daily PO, 6 days					
Nanki *et al.* [1]	53/F	Systemic lupus erythematosus	P	20 mg daily; PO, (105 days)	658/871	ND a	Macrovesicular steatosis and mild periportal PMN a infiltration (autopsy)	None	Death
Dourakis *et al.* [2]	67/F	Dermatomyositis	P	25 mg/t.i.d. IV, (26 days)	545/1229	2092/467	Macrovesicular steatosis and mild portal lymphocyte and PMN infiltration (autopsy)	None	Death
Weissel *et al.* [3]	71/F	Graves ophthalmopathy	MP + C a	1,0 g daily IV, 3 days- tapering to 0 within 10-14 days; 5 courses	ND	ND	Necrosis of liver parenchyme (autopsy)	Methimazole started 6 months before MP, continued until the last course	Death
Salvi *et al.* [17]	43/F	Thyroid associated ophthalmopathy	MP	7,5 mg/kg IV, every 2 weeks (4 courses)	1200/850	ND	Compatible with autoimmune hepatitis	Levothyroxine since 3 years	Normalization of liver tests
Hofstee *et al.*[18]	46/F	Multiple sclerosis	MP	0,5 g IV, 5 days	N	ND	Biopsy not done	None	Normalization of liver tests after MP discontinuation
				1 g IV, 3 days; 2 years later	1095/755	156/140			
				1 g IV, 3 days; 4 years later	1600/900	ND			
				1 g IV, 3 days ; 9 years later	2350/950	ND			
Das *et al. *[6]	48/F	Multiple clerosis	MP	ND; 3 courses	1650/1430	ND/590	Preserved architecture with lobular infiltration by lymphocytes, eosinophils and plasma cells	None	Normalization of liver tests after MP discontinuation
Topal *et al.* [19]	47/F	Vasculitis of the central nervous system	MP	ND; PO, 7 days course	2478/1600	242/138	Biopsy not done	Topiramate; since 1 year and during MP course	Normalization of liver tests after MP discontinuation
Rivero Fernandez *et al.* [4]	57/F	Multiple sclerosis	MP	1,0 g IV, 3 days	1223/543	71/113	Acute necrotic hepatitis with ceroid-laden macrophage hyperplasia	None	Normalization of liver tests 3 months after MP discontinuation
Takahashi *et al.* [7]	43/F	Multiple sclerosis	MP+P	1,0 g IV, 3 days followed by 50 mg/dL PO, for 1 month	Normal	Normal	Bridging perivenular necrosis with infiltration by inflammatory cells including eosinophils (first biopsy)	None	
			MP+P	1 g IV, 3 days; 3 years later followed by 50 mg/d PO, plus	1067/1102	26/377	Bridging perivenular necrosis and interface hepatitis (second biopsy)	6 doses of Interferone beta-1b	Normalization of liver tests several months after MP discontinuation. P tapered within 8 months
			MP	1 g IV, 3 days; 5 days later					Normalization of liver tests 3 months after MP discontinuation. P tapered within 3 months
			MP	1 g IV, 3 days; 13 monts late	566/875	1785/214			
Loraschi *et al.* [5]	33/M a	Demyelinating encephalopathy	MP	Total dose 2,5 g IV, 4 days course	1042/349	ND/ND	Focal liver cell necrosis in acinar zones 2 and 3, monocyte/macrophage infiltration, Kupffer cell hyperplasia, acidophilic bodies and focal microvesicular steatosis	None	Normalization of liver tests 20 days after MP discontinuation
Loraschi *et al.* [5]	27/F	Retrobulbar optic neuritis	MP	Total dose 4,5 g IV, 6 days course	122/39	ND/ND	Biopsy not done	None	Normalization of liver tests 4 days after MP discontinuation
Gutkowski *et al.*	24/F	Multiple sclerosis	MP	Total dose 3,0g IV, 6 days	Normal	Normal	Biopsy not done	None	
(present case)				course					
			MP	Total dose 3,0g IV, 6 days course; 6 weeks later	1740/900	186/186		4 doses of Interferone beta-1b 0,5 g acetami- nophen; 4 times	Normalization of liver tests 3 weeks after MP discontinuation
			MP	Total dose 3,0g IV, 6 days course; 3 months later	1129/1488	168/164		None	Normalization of liver tests 6 weeks after MP discontinuation

^a^ Abbereviations: ALP, alkaline phosphatase; ALT, alanine aminotransferase; AST, aspartate aminotransferase; C, cortisone; F, female; GGT, γ- glutamyltransferase; M, male; MP, methylprednisolone; N, normal; ND, not determined; P, prednisolone; PMN, polymorphonuclear leukocyte.

The clinical course of liver injury varied from asymptomatic hypertransaminasemia to fulminant hepatic failure (3 deaths). In vivo liver histopathology was performed in only 8 cases, showing a large spectrum of lesions. Fernandez et al. reported recurrent acute hepatitis that was characterized by necrosis on histopathological examination, related to intravenous MT that was given on 3 occasions for the management of relapsing multiple sclerosis [4]. Loraschi et al. reported 2 cases of liver damage that was related to high-dose MT therapy for demyelinating disease [5]. The first patient, a 33-year-old man, developed a histologically recognized acute steatohepatitis 5 weeks after last exposure to MT. The second patient, a 27-year-old woman, presented with moderate and asymptomatic augmentation of liver enzymes 6 days after withdrawal of MT. Hypersensitivity reactions were not observed in any patient. Despite their anti-inflammatory and antiallergic properties, corticosteroids also trigger immunoallergic liver injuries. Das et al. reported recurrent liver injuries that occurred 6 weeks and 3 weeks following the second and third course of intravenous MT, respectively, for multiple sclerosis [6]. Liver biopsy showed lobular, primarily perivenular, infiltration with activated lymphocytes, eosinophils, and plasma cells. Moreover, Japanese authors reported the occurrence of autoimmune hepatitis, confirmed by liver histology, in a patient with multiple sclerosis who was treated with MP pulses [7]. In their opinion, autoimmune hepatitis was a consquence of an immune rebound phenomenon after pulsed MP.

The mechanisms of corticosteroid-induced liver injury are unclear and only occasionally are related to reactivation of HBV infection or to the excipient of the MT preparation [6][8][9]. Though low doses of corticosteroids are considered safe for the liver, chronic administration of these drugs may be associated with steatosis or steatohepatitis [2][10]. Intrinsic hepatotoxicity of high doses of corticosteroids is rather unlikely, as serious hepatic injuries that are related to MT occur rarely and are unpredictable. The majority of hepatotoxic drugs causes idiosyncratic reactions. There are two types of idiosyncrasy: immunoallergic and nonallergic (metabolic). The essence of an immunoallergic reaction is a complex interaction between a parent drug or its metabolites with immunologically competent cells, leading to necrosis and apoptosis of hepatocytes. Released cytokines additionally damage liver cells or have immune-modulating effects [11]. In metabolic idiosyncrasy, the liver injury is caused by aberrant hepatic metabolism, leading to overproduction of reactive metabolites from a parent compound. In general, idiosyncratic drug-induced hepatotoxicity is considered to be unpredictable and dose-independent [11]; however, it has been suggested that there is dose-dependency for drugs that are extensively metabolized in the liver [12]. MT is metabolized by cytochrome P450 3A4 (CYP3A4), and its metabolites undergo renal elimination.

In our patient, we did not observe any systemic hypersensitivity symptoms before or during liver injury. After two exposures to MT, the hepatitis episodes appeared 4 weeks after drug withdrawal. In metabolic idiosyncrasy, the latency periods vary considerably from days to months, and adverse reactions can occur even several weeks after drug discontinuation. Such examples are not common but have been reported for some antibiotics, such as amoxicillin clavulanate, midecamycin, trovafloxacin, and flucloxacilline [13][14][15][16]. The duration of anti-inflammatory effects after a single intramuscular injection of 40-80 mg MT ranges from 4 days to 8 days. Thus, it seems unlikely that chemically active metabolites damage hepatocytes 4 weeks after drug discontinuation. Rather, this scenario suggests a delayed immune response to the metabolite that is bound to the host protein and successive presentation as a neoantigen to the immune cells following the death of hepatocytes. The appearance of SMA following exposure to MT might be a feature of the immunological response.

## 4. Conclusion

MT pulses are increasingly used by neurologists, rheumatologists, and endocrinologists to treat various autoimmune diseases. The general awareness of the potential hepatotoxicity of high-dose corticosteroids is very low. Corticosteroid-induced liver injury may occur as acute hepatitis that develops several weeks after short-term drug exposure. We therefore feel that MT should be placed on the list of hepatotoxic drugs and that patients who receive corticosteroid pulses should be screened for potential liver injury.
